# The association between socioeconomic position and the symptoms and concerns of hospital inpatients seen by specialist palliative care: Analysis of routinely collected patient data

**DOI:** 10.1177/02692163221115331

**Published:** 2022-08-10

**Authors:** Joanna M Davies, Katherine E Sleeman, Christina Ramsenthaler, Wendy Prentice, Matthew Maddocks, Fliss EM Murtagh

**Affiliations:** 1 Cicely Saunders Institute of Palliative Care, Policy and Rehabilitation, King’s College London, London, UK; 2 King’s College Hospital NHS Foundation Trust, London, UK; 3 Faculty of Health Sciences, Institute of Nursing, Zurich University of Applied Sciences, Winterthur, Switzerland; 4 Wolfson Palliative Care Research Centre, Hull York Medical School, University of Hull, Hull, UK

**Keywords:** Socioeconomic factors, health equity, symptom assessment, palliative care

## Abstract

**Background:**

Understanding how socioeconomic position influences the symptoms and concerns of patients approaching the end of life is important for planning more equitable care. Data on this relationship is lacking, particularly for patients with non-cancer conditions.

**Aim:**

To analyse the association between socioeconomic position and the symptoms and concerns of older adult patients seen by specialist palliative care.

**Design:**

Secondary analysis of cross-sectional, routinely collected electronic patient data. We used multivariable linear regression with robust standard errors, to predict scores on the three subscales of the Integrated Palliative care Outcome Scale (IPOS; physical symptoms, emotional symptoms and communication and practical concerns) based on patient level of deprivation, measured using Index of Multiple Deprivation.

**Setting/participants:**

Consecutive inpatients aged 60 years and over, seen by specialist palliative care at two large teaching hospitals in London between 1st January 2016 and 31st December 2019.

**Results:**

Seven thousand eight hundred and sixty patients were included, 38.3% had cancer. After adjusting for demographic and clinical characteristics, patients living in the most deprived areas had higher (worse) predicted mean scores on the communication and practical subscale than patients living in the least deprived areas, 5.38 (95% CI: 5.10, 5.65) compared to 4.82 (4.62, 5.02) respectively. This effect of deprivation diminished with increasing age. Deprivation was not associated with scores on the physical or emotional symptoms subscales.

**Conclusions:**

Targetting resources to address practical and communication concerns could be a strategy to reduce inequalities. Further research in different hospitals and across different settings using patient centred outcome measures is needed to examine inequalities.

**What is already known about the topic?**

- Research on socioeconomic inequality towards the end of life has tended to focus on inequality in access to care.
- A small number of studies, only on patients with advanced cancer, suggest that people with lower socioeconomic position may experience worse pain, anxiety, depression, overall symptom burden, and have poorer emotional well-being and quality of life.

**What this paper adds?**

- This evaluation of routinely collected data on all inpatients seen by specialist palliative care at two large London hospitals between 2016 and 2019, finds that patients who lived in more deprived areas had worse communication and practical concerns at initial assessment.
- The difference in communication and practical concerns according to level of deprivation held after adjusting for multiple clinical and demographic characteristics.
- The effect of deprivation on communication and practical concerns diminished with increasing age and was not statistically significant for patients aged >83 years.
- Patient level of deprivation was not associated with physical or emotional symptoms.

**Implications for practice theory or policy**

- This study provides novel and practical insights into the relationship between area-based deprivation and the symptoms and other concerns of hospital inpatients seen by palliative care.
- The findings indicate that targetting resources to address practical and communication concerns could be a strategy to reduce inequalities for people approaching the end of life.

## Background

In high income countries, end-of-life hospital admissions, death in hospital (compared to home or hospice) and a lack of access to specialist palliative care are consistently more common for people with lower socioeconomic position. Research on socioeconomic inequality towards the end of life has tended to focus on inequality in access to care.^1,2^ There is a lack of data on how socioeconomic position influences the symptoms and concerns of people towards the end of life.^3^

Patient-centred outcome measures are validated questionnaires that measure the health status, symptoms and well-being of patients.^4^ These measures are increasingly used in palliative and end-of-life care for research, quality improvement and in routine care.^5,6^ Understanding how social factors such as age, deprivation and ethnicity are associated with the symptoms and concerns of patients with advanced illness is important for planning the delivery of more equitable care. This challenge is particularly important in the context of an ageing population^7^ and increasing social inequality.^8,9^

Existing evidence, based on a small number of studies and only on patients with advanced cancer, suggests that people with lower socioeconomic position may experience worse pain, anxiety, depression and overall symptom burden,^10–12^ and have poorer emotional well-being and quality of life.^13^ More studies including patients with non-cancer conditions are needed to strengthen the evidence on socioeconomic inequality in the symptom burden of dying patients.

This study aims to analyse the association between socioeconomic position and the symptoms and other concerns of older adult patients seen by specialist palliative care at two large London-based teaching hospitals between 2016 and 2019. Based on existing evidence,^10–13^ we hypothesised that the symptoms and concerns of patients would be worse for patients living in more deprived areas.

## Methods

### Study design, data source, setting and participants

Secondary analysis of cross-sectional, electronic patient data, including all older adult (aged 60 or older) inpatients seen by specialist palliative care (first episode of care only) at two large teaching hospitals in London between 1st January 2016 and 31st December 2019. We focussed on older adults because their symptoms and concerns are likely to be different to those of younger adults.^14–16^ At the time of data collection, the multi-professional specialist palliative care team provided an advisory service to both hospitals, comprised of a visiting service 09:00–17:00 Monday to Friday, with 24/7 consultant-led telephone support, and a limited weekend and public holiday visiting service.^17^ Both hospitals have emergency departments, acute medical beds and intensive care units. Hospital 1 is situated on the outskirts of the city and has 512 beds, hospital 2 has 1100 beds and serves an inner-city population.^17^

### Outcome and exposure variables

The outcome variables were the three subscales of the Integrated Palliative care Outcome Scale (IPOS): physical symptoms, emotional symptoms, communication/practical issues (Table 1).^18^ IPOS is a brief, validated, patient self-reported and staff proxy-reported outcome measure, used to assess symptoms and concerns in advanced illness.^18^ The IPOS was first introduced into routine clinical care in both hospitals in 2016 and is part of the electronic patient record. The IPOS was completed by clinical staff up to 3 days after the first clinical assessment. The IPOS asks about how much the patient has been affected by symptoms and other concerns over the last 3 days, higher scores indicate worse symptoms or concerns. Subscales scores were summed from the item scores. For cases with at least half the items complete for the physical and emotional subscales, and at least one item complete for the practical subscale, the subscale median score from the non-missing items was imputed for missing items.

**Table 1.** summary of the Integrated Palliative care Outcome Scale (IPOS) subscales.

IPOS subscale	# items	Score range	Items
Physical symptoms	10	0–40	Pain
			Shortness of breath
			Weakness or lack of energy
			Nausea
			Vomiting
			Poor appetite
			Constipation
			Score or dry mouth
			Drowsiness
			Poor mobility
Emotional symptoms	4	0–16	Patient anxiety
			Family anxiety
			Depression
			Feeling at peace
Communication/practical concerns	3	0–12	Sharing feelings
			Information needs
			Practical matters

The main exposure was a national area-based measure of socioeconomic position, the Index of Multiple Deprivation (IMD) for England (2019).^19^ Patient postcodes were linked to lower super output area (LSOA) codes which were linked to the IMD. IMD was summarised using national quintile groups (quintile 1 is most deprived). Missing or erroneous postcodes, or postcodes outside of England were expected in a small proportion of cases (<5%) and excluded from the study.

### Analysis

We selected the following covariates based on existing knowledge^10,18,20^: age, gender, ethnicity, living alone, diagnosis, palliative Phase of Illness at initial assessment,^21^ Australia-modified Karnofsky Performance Status (AKPS)^22^ at initial assessment, and hospital site. Variables are described for the overall population and separately for each deprivation group, using standard descriptive statistics. To maintain sample size, missing data on the ethnicity and living alone variables were coded as separate categories and included in the modelling. This limits the interpretation of the effects for ethnicity and living alone but supports the main purpose of their inclusion as confounders of the relationship between deprivation and IPOS. We used ordinary least squares, multiple linear regression models and applied robust standard errors to account for violations of normality assumptions in the residuals.^23^ Deprivation was treated as an ordered categorical variable to allow for non-linearity in the relationship. We compared a minimally adjusted model that controlled only for age, sex, hospital site and deprivation, with a model that controlled for all other covariates, using *R*^2^ and Wald *F* statistics. For the main model we present unstandardised coefficients and predicted mean scores for each deprivation group, and use the standardised mean difference to derive a Cohen’s d effect size.^24^ Statistical significance was set a priori at *p* < 0.05 with no adjustment for multiplicity.

### Moderation by age and gender

The effect of socioeconomic position on health towards the end of life may diminish with increasing age,^25^ gender may also moderate social determinants of health^8,9^ and influence end-of-life care.^26^ To investigate moderation by age and gender we included interaction effects in our model and compared these models against the main model. We plotted the linear effect of deprivation on subscale scores across the age range to help interpret interaction effects.

### Sensitivity analysis

We repeated the main model using a complete case analysis with missing data on the subscales handled listwise. We also repeated the main analysis on a dataset where missing IPOS items were imputed based on all other variables, using semi-parametric predictive mean matching chained multiple imputation, with 40 sets, proportionate to the amount of missing data.^27–30^ Given the limitations of using multiple imputation for outcome variables and the potential for missing not at random mechanisms in our data, we chose to use the multiple imputation as a sensitivity analysis rather than for our main analysis.^31^

To evaluate the potential for unmeasured confounders, we report e-values; defined as the minimum strength of association on the risk ratio scale that an unmeasured confounder would need to have with both the exposure and the outcome to fully explain away the specific effect, in our case the main effect of deprivation on the IPOS subscales, conditional on the covariates.^32^

All analysis, including sensitivity analysis, was pre-specified and carried out in Stata (version 17); the analytical code and analysis plan is available from: https://github.com/joannamariedavies.

### Results

After excluding 103 (1.3%) patients with missing or erroneous postcode information, the sample included 7860 patients (Table 2). Compared to national data on deaths in England and Wales in 2019, the sample was less deprived and had a larger proportion of cancer diagnoses (Supplemental Tables 1 and 2). Hospital 2 cared for more patients in deprived areas and had a younger population compared to hospital 1 (Supplemental Table 3). The main reasons for referral to specialist palliative care, were for pain or other physical symptoms (34.1%) or terminal care (31.6%) (Supplemental Table 4). About half of the sample were discharged by specialist palliative care at the end of the episode of care, into the community, to hospice or remaining in hospital; 3993 (50.8%) died during the episode of care. Date of death was available for 3953 patients and was a median (IQ range) of 3 (1–6) days after the first clinical assessment.

**Table 2.** Patient characteristics by patient level of area-based deprivation, (*n* and column percentage, unless otherwise stated).

	Overall	Area-based level of deprivation, quintile groups (q1 is most deprived)				
		q1	q2	q3	q4	q5
*N*	7860	1145 (14.6%)	1775 (22.6%)	1318 (16.8%)	1853 (23.6%)	1769 (22.5%)
Age						
Median (IQ range)	82 (74, 89)	81 (72, 87)	81 (71, 87)	82 (72, 88)	84 (76, 90)	84 (76, 89)
Missing	11 (0.1%)	3 (0.3%)	3 (0.2%)	3 (0.2%)	1 (0.1%)	1 (0.1%)
Gender						
Women	4121 (52.4%)	619 (54.1%)	934 (52.6%)	657 (49.8%)	1000 (54.0%)	911 (51.5%)
Missing	1 (<1%)	0 (0.0%)	1 (0.1%)	0 (0.0%)	0 (0.0%)	0 (0.0%)
Ethnicity						
White British	5226 (66.5%)	631 (55.1%)	991 (55.8%)	815 (61.8%)	1394 (75.2%)	1395 (78.9%)
White other	370 (4.7%)	80 (7.0%)	94 (5.3%)	74 (5.6%)	77 (4.2%)	45 (2.5%)
Black	627 (8.0%)	194 (16.9%)	264 (14.9%)	112 (8.5%)	43 (2.3%)	14 (0.8%)
Asian	174 (2.2%)	22 (1.9%)	56 (3.2%)	27 (2.0%)	37 (2.0%)	32 (1.8%)
Other	223 (2.8%)	42 (3.7%)	81 (4.6%)	51 (3.9%)	30 (1.6%)	19 (1.1%)
Missing	1240 (15.8%)	176 (15.4%)	289 (16.3%)	239 (18.1%)	272 (14.7%)	264 (14.9%)
Living alone						
Yes (versus not alone)	2220 (28.2%)	345 (30.1%)	463 (26.1%)	377 (28.6%)	508 (27.4%)	527 (29.8%)
Missing	1491 (19.0%)	237 (20.7%)	398 (22.4%)	285 (21.6%)	300 (16.2%)	271 (15.3%)
Diagnosis						
Cancer	3013 (38.3%)	421 (36.8%)	653 (36.8%)	490 (37.2%)	712 (38.4%)	737 (41.7%)
Dementia	642 (8.2%)	103 (9.0%)	163 (9.2%)	93 (7.1%)	148 (8.0%)	135 (7.6%)
Cardiovascular	1351 (17.2%)	186 (16.2%)	327 (18.4%)	253 (19.2%)	313 (16.9%)	272 (15.4%)
Respiratory	460 (5.9%)	77 (6.7%)	130 (7.3%)	85 (6.4%)	78 (4.2%)	90 (5.1%)
Other	2222 (28.3%)	327 (28.6%)	469 (26.4%)	369 (28.0%)	563 (30.4%)	494 (27.9%)
Missing	172 (2.2%)	31 (2.7%)	33 (1.9%)	28 (2.1%)	39 (2.1%)	41 (2.3%)
Phase of Illness at initial assessment						
Stable	343 (4.4%)	70 (6.1%)	73 (4.1%)	51 (3.9%)	81 (4.4%)	68 (3.8%)
Unstable	2904 (36.9%)	451 (39.4%)	726 (40.9%)	536 (40.7%)	631 (34.1%)	560 (31.7%)
Deteriorating	1682 (21.4%)	215 (18.8%)	344 (19.4%)	272 (20.6%)	396 (21.4%)	455 (25.7%)
Dying	2514 (32.0%)	355 (31.0%)	528 (29.7%)	390 (29.6%)	647 (34.9%)	594 (33.6%)
Missing	417 (5.3%)	54 (4.7%)	104 (5.9%)	69 (5.2%)	98 (5.3%)	92 (5.2%)
AKPS* at initial assessment						
Mean (SD)	25.8 (16.0)	26.1 (16.2)	26.4 (16.4)	27.0 (16.5)	24.6 (15.5)	25.3 (15.6)
Missing	1514 (19.3%)	216 (18.9%)	333 (18.8%)	252 (19.1%)	359 (19.4%)	354 (20.0%)
Site						
Hospital 1	4392 (55.9%)	377 (32.9%)	515 (29.0%)	537 (40.7%)	1413 (76.3%)	1550 (87.6%)
Hospital 2	3468 (44.1%)	768 (67.1%)	1260 (71.0%)	781 (59.3%)	440 (23.7%)	219 (12.4%)

* AKPS: Australia-modified Karnofsky Performance Status

Weakness or lack of energy, and poor mobility were the most common physical symptoms. Family anxiety (on the emotional subscale) and practical matters (on the communication/practical subscale) were the most prevalent concerns overall (Supplemental File Figure 1 and Table 5).

Table 3 describes the distribution of the IPOS subscale scores for the complete cases and following median imputation. Following median imputation, deprivation was not statistically significantly associated with missing data on the communication/practical or emotional subscales. On the physical subscale, patients in deprivation quintiles q3 and q4 had higher odds of having missing data compared to the least deprived group (q5) (Supplemental Table 6). Patients who had a cancer diagnosis, those who spent more time with a clinician during their episode of care, and those in hospital 1, were less likely to have missing IPOS data. Missing data on the ethnicity and living alone variables was between 11.3% and 14.3% (Supplemental Table 7).

**Table 3.** Summary of Integrated Palliative care Outcome Scale (IPOS) subscales: complete cases, and imputed cases.*

*N* = 7860	Physical subscale	Emotional subscale	Communication/Practical subscale
Complete cases, *n* (%)	3863 (49.1%)	1795 (22.8%)	2637 (33.6%)
Mean (sd)	7.9 (5.5)	4.7 (3.5)	4.9 (3.2)
Median (IQ range)	7 (3–12)	4 (2–7)	5 (2–8)
Complete and imputed cases*, *n* (%)	4883 (62.1%)	4690 (59.7%)	4961 (63.1%)
Mean (*sd*)	9.1 (6.5)	5.0 (3.7)	5.1 (3.6)
Median (IQ range)	8 (4–13)	4 (2–8)	5 (3–8)

* For cases with number of missing items <6 for physical subscale, <3 for emotional subscale, <3 for communication/practical subscale, the median score for the non-missing items was imputed to the missing items

## Results from the main model

**Figure 1.**
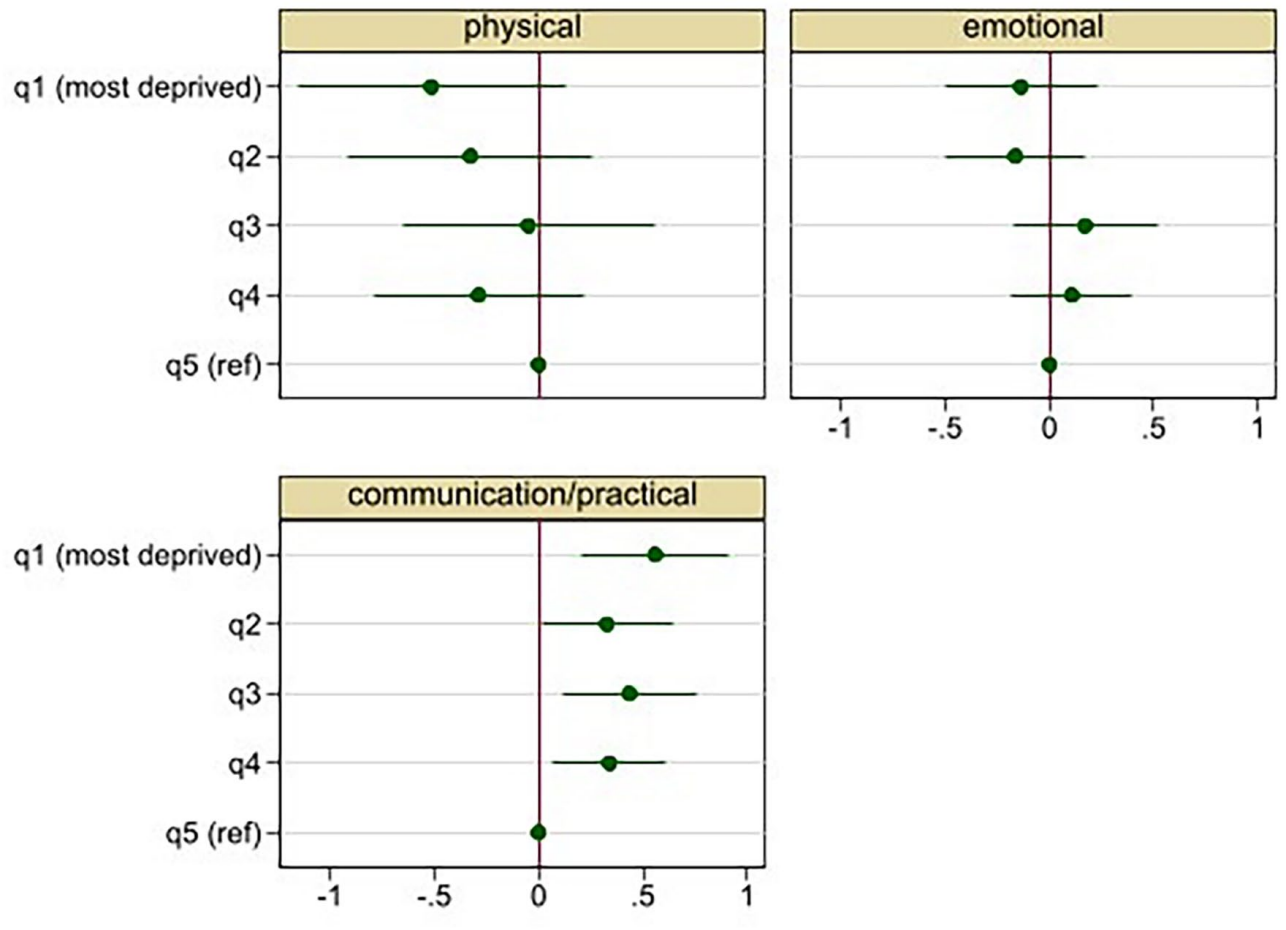
Adjusted* regression coefficients and 95% CI for the association between patient level of area-based deprivation and Integrated Palliative care Outcome Scale (IPOS) subscales. *Analysis adjusted for: age, sex, ethnicity, living alone, diagnosis, Phase of Illness, Australia-modified. Karnofsky Performance Status and hospital site

Patients living in the most deprived areas (q1) had statistically significantly higher (worse) scores on the communication/practical subscale than patients living in the least deprived areas (q5) (Figure 1 and Table 4, and Supplemental Table 8). The adjusted predicted mean score on the communication/practical subscale for patients in q1 was 5.38 (95% CI 5.10, 5.65), compared to 4.82 (4.62–5.02) for those in q5. The standardised mean difference between q1 and q5 was 0.16 (95% CI 0.07–0.23) indicating a small effect size. The results suggest a roughly linear relationship with an increase in communication and practical concerns for each increase in deprivation (Figure 1 and Table 4). Deprivation was not associated with scores on the physical or emotional subscales.

**Table 4.** Adjusted* association between patient level of area-based deprivation and Integrated Palliative care Outcome Scale (IPOS) subscale scores.

	Physical subscale			Emotional subscale			Communication/Practical subscale		
	Adjusted coeff. [95% CI]	Predicted mean (sd) score [95% CI]	E value	Adjusted coeff. [95% CI]	Predicted mean (sd) score [95% CI]	E value	Adjusted coeff. [95% CI]	Predicted mean (sd) score [95% CI]	E value
q1 (most deprived)	−0.51 [−1.15, 0.12]	8.78 [8.29, 9.27]	1.36	−0.14 [−0.50, 0.23]	4.83 [4.56, 5.11]	1.22	0.56 [0.21, 0.91]	5.38 [5.10, 5.65]	1.57
q2	−0.33 [−0.91, 0.26]	8.97 [8.56, 9.38]	1.27	−0.16 [−0.50, 0.17]	4.81 [4.58, 5.03]	1.25	0.33 [0.01, 0.64]	5.15 [4.92, 5.37]	1.39
q3	−0.05 [−0.65, 0.56]	9.25 [8.79, 9.71]	1.09	0.17 [−0.17, 0.52]	5.14 [4.88, 5.40]	1.25	0.44 [0.12, 0.76]	5.25 [5.01, 5.50]	1.47
q4	−0.29 [−0.79, 0.22]	9.01 [8.65, 9.37]	1.25	0.11 [−0.19, 0.40]	5.08 [4.87, 5.29]	1.19	0.34 [0.07, 0.61]	5.16 [4.96, 5.35]	1.4
q5 (least deprived)	ref	9.29 [8.92, 9.67]	ref	Ref	4.97 [4.75, 5.19]	ref	ref	4.82 [4.62, 5.02]	ref

* Adjusted for: age, sex, ethnicity, living alone, diagnosis, Phase of Illness, Australia-modified Karnofsky Performance Status, hospital site

* Results in table 4 focus on the main exposure of interest (deprivation) and not on the other covariates,^33^ model results for all covariates are available in the Supplemental Table 8.

### Moderation by age and gender

Interaction effects between age and deprivation were statistically significant for the communication/practical subscale and not statistically significant for the physical and emotional subscales (Supplemental File, Table 9, model 3). Gender and deprivation interaction effects were not statistically significant for any subscale (Supplemental File, Table 9, model 4).

**Figure 2.**
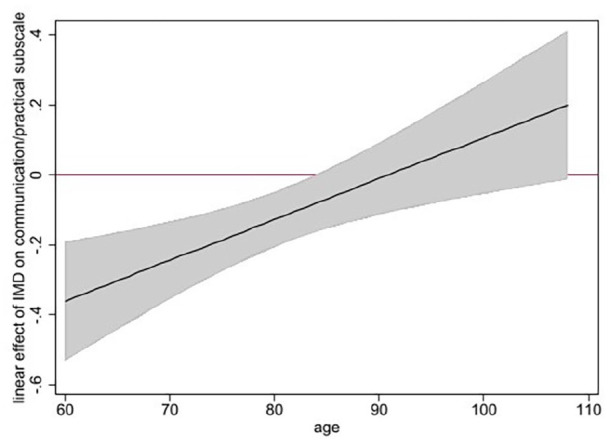
Adjusted* linear effect and 95% CI of patient level of area-based deprivation on the Integrated Palliative care Outcome Scale (IPOS) communication/practical subscale, moderated by age (figure shows that the negative effect of being less deprived on the communication/practical subscale is stronger at younger ages and not statistically significant after age 83 years) *analysis adjusted for: age, sex, ethnicity, living alone, diagnosis, Phase of Illness, Australia-modified Karnofsky Performance Status and hospital site.

Figure 2 shows that the negative effect of living in a less deprived area on communication and practical issues, is stronger at younger ages and not statistically significant for people aged >83 years.

### Sensitivity analysis

The complete case and multiple imputation analysis had similar results to the main analysis (Supplemental File, Table 10). The low e-values (Table 4) suggest a high likelihood that unmeasured confounders exist that could, if included in the model, explain away the effects of deprivation.

## Discussion/conclusion

### Main findings

In this evaluation of routine data on older adult inpatients seen by specialist palliative care at two large London hospitals, patients who lived in more deprived areas had worse communication and practical concerns at initial assessment. The difference in communication and practical concerns between patients living in the least and most deprived areas was small in effect size, but the results suggest a trend towards a step-wise social gradient that held after adjusting for multiple clinical and demographic characteristics. The effect diminished with increasing age and was not statistically significant for patients over 83 years old. Area-based deprivation was not associated with physical or emotional symptoms.

### What this study adds

There is growing evidence that people with lower socioeconomic position face additional challenges towards the end of life, including problems with inadequate housing,^3,34^ fuel poverty^35^ and loss of earnings,^36,37^ and may have different communication needs.^10,38^ Our finding that hospital inpatients who live in more deprived areas present to specialist palliative care with worse communication and practical concerns is consistent with this evidence and reflects wider structural inequalities in society. The effect of deprivation in our analysis was small, yet this inequality potentially impacts large numbers of patients and could be modifiable. Increasing financial support for patients and families dealing with terminal illness,^39^ improving cultural competency training for health care professionals and making resources available in multiple languages and for different literacy levels have been cited as ways to address inequality at the end of life.^40^ The findings from our study support calls for a better understanding of how socioeconomic inequality impacts needs towards the end of life.^3^

Our analysis did not investigate the effect of deprivation on the separate IPOS items, therefore we don’t know which aspects of communication and practical concerns drive the effect we observed. Qualitative work with patients living in deprived areas, to understand the type of practical problems faced, and whether communication needs relate to language, literacy, or other barriers, could help to identify ways to better meet patient needs.

In contrast to earlier studies of patients with advanced cancer,^10–12^ we did not find that deprivation was associated with the physical or emotional symptoms of hospital inpatients seen by specialist palliative care. This finding contrasts with the global evidence on the association between socioeconomic position and physical and mental health in the general population^41^ and with our earlier analysis that found that worse health partly explained why people with lower wealth had more hospital admissions in the last 2 years of life.^25^ Several factors may explain these contrasting results.

First, our sample is limited to patients who received specialist palliative care. Mortality bias, and bias in referrals, is likely to mean that a disproportionately higher number of people with lower socioeconomic position are never referred to palliative care, or die from sudden causes before reaching hospital.^42,43^ This potentially biases our results through an underrepresentation of people living in the most deprived areas. Comparison with national death registration data suggests that our sample was less deprived and had a larger proportion of cancer patients than the national end-of-life population (Supplemental File, Tables 1 and 2).

Second, in this observational study unmeasured service or regional-specific confounders are important to consider.^17,44^ For example, the quality of the symptom control received by patients on hospital wards prior to referral to palliative care is likely to vary between hospitals and wards and potentially moderates the effect of deprivation on symptoms. There are also growing regional differences in health inequalities in the UK, for example, life expectancy is lowest in the North East and highest in London.^8^ Further studies on patients in different hospitals, in different regions, and on patients in community settings are needed to strengthen the evidence.

Third, we cannot rule out measurement bias in our outcome measure. IPOS is a validated and widely used tool^18^ and the difference we detected for groups based on deprivation in the communication/practical subscale, suggests that IPOS is sensitive to socioeconomic difference. However, the IPOS questions could be biased towards capturing symptoms and problems that are more relevant to people with higher socioeconomic position. Staff reported measures could also be less good at identifying symptoms in patients with lower socioeconomic position. There may be differences in the way that patients perceive and manage symptoms that could influence professional assessments of pain and other symptoms.^45^ Implicit class and other biases including racial bias^40^ in health care workers could also limit the effectiveness of the tool, for example in the UK, only 4% of doctors^46^ and 43% of nurses^47^ are from low socioeconomic position backgrounds. Further validation of staff and patient inter-rater reliability of the IPOS and content-validity for different social, economic, and ethnic groups would strengthen the tool.

We found that the effect of deprivation on communication and practical concerns diminished with increasing age. This could reflect bias in the sample, an under-representation of more socioeconomically deprived older adults, or an under-representation of care home residents who are less likely to be admitted to hospital^48^ and may be more socioeconomically deprived.^49^ Cohort effects, for example less wealth inequality among older adults due to the relatively generous state pension,^39^ could also limit the effect of deprivation on communication and practical concerns for older people.

### Strengths and limitations

This service evaluation has extended the evidence on socioeconomic inequality in the symptoms and concerns of patients towards the end of life to a large and diverse population of hospital inpatients. The work demonstrates the utility of linking area-based deprivation information to routinely collected patient centred outcome measures for understanding socioeconomic differences in the needs of patients.

We used an area-based measure of deprivation which is limited by the ecological fallacy (the attempt to infer something about an individual from aggregate data). A further limitation of basing deprivation score on the postcode of residence is that for care home residents this may be a poor reflection of socioeconomic position. However, area-based measures constructed using small, homogenous geographies offer a high quality and convenient way to link socioeconomic position into routine data sources when individual level measures are not available. Area-based measures that combine multiple domains of deprivation such as the Index of Multiple Deprivation, may capture more variance in socioeconomic position than individual level items such as education.^50^

There was a high proportion of missing data on the IPOS subscales. There was no clear relationship between missing data on the subscales and our main exposure of deprivation which suggests that our main effects should be robust. However, we cannot discount that the missing data might bias our results. In this routinely collected data, the amount of missing data was comparable to a prospective longitudinal study on a similar topic, where data was collected on 65% of patients eligible for inclusion.^10^ In our study, fewer items on the physical subscale were missing, potentially indicating staff preference towards completing items about physical symptoms. Missing IPOS data was associated with less clinical time with the patient (Supplemental Table 6). More work is needed to set guidelines on the proportion of missing data expected in the routine collection of IPOS data in different settings and for the different items.

A strength of our analysis is the use of robust sensitivity analysis using multiply imputed data and complete case analysis to address the missing data. The high proportion of missing ethnicity information in our data, typical in hospital data from the UK,^51^ limits what we can learn about ethnicity effects in this study. More work is needed to investigate the intersection between ethnicity and socioeconomic position in palliative and end-of-life care research, an important aspect of this is improving the collection of self-reported ethnicity data in hospital data.^40^

## Conclusion

In this evaluation of inpatients seen by specialist palliative care at two large London hospitals, patients living in more deprived areas had worse communication and practical concerns at first assessment. This indicates that targetting resources to address practical and communication concerns could be a strategy to reduce inequalities. We did not find that deprivation was associated with physical or emotional symptoms. This could reflect a lack of association or potential sample bias, unmeasured confounders, measurement bias or missing data. Further research is needed to understand the impact of communication and practical concerns on other outcomes including place of death and hospital admissions, and to investigate socioeconomic inequality in the symptoms and concerns of patients in different hospitals and in different settings.

## Supplemental Material

sj-pdf-1-pmj-10.1177_02692163221115331 – Supplemental material for The association between socioeconomic position and the symptoms and concerns of hospital inpatients seen by specialist palliative care: Analysis of routinely collected patient data Supplemental material, sj-pdf-1-pmj-10.1177_02692163221115331 for The association between socioeconomic position and the symptoms and concerns of hospital inpatients seen by specialist palliative care: Analysis of routinely collected patient data by Joanna M Davies, Katherine E Sleeman, Christina Ramsenthaler, Wendy Prentice, Matthew Maddocks and Fliss EM Murtagh in Palliative Medicine

## References

[1] Davies JM Sleeman KE Leniz J , et al. Socioeconomic position and use of healthcare in the last year of life: a systematic review and meta-analysis. PLoS Med 2019; 16: e1002878–e1002782. 10.1371/journal.pmed.1002782 PMC6478269 31013279

[2] French M Keegan T Anestis E , et al. Exploring socioeconomic inequities in access to palliative and end-of-life care in the UK: a narrative synthesis. BMC Palliat Care 2021; 20: 188. 34802450 10.1186/s12904-021-00878-0 PMC8606060

[3] Rowley J Richards N Carduff E , et al. The impact of poverty and deprivation at the end of life: a critical review. Palliat Care Soc Pract 2021; 15: 26323524211033873. 34541536 10.1177/26323524211033873 PMC8442481

[4] Deerberg-Wittram J Guth C Porter ME . Value-based competition: the role of Outcome Measurement. Public Health Forum 2013; 21: 12–13.

[5] Bausewein C Le Grice C Simon S , et al. The use of two common palliative outcome measures in clinical care and research: a systematic review of POS and STAS. Palliat Med 2011; 25: 304–313. 21464119 10.1177/0269216310395984

[6] Collins ES Witt J Bausewein C , et al. A systematic review of the use of the palliative care outcome scale and the support team assessment schedule in Palliative Care. J Pain Symptom Manag 2015; 50: 842–NaN53.e19. 10.1016/j.jpainsymman.2015.07.015 26335764

[7] Christensen K Doblhammer G Rau R , et al. Ageing populations: the challenges ahead. Lancet 2009; 374: 1196–1208. 19801098 10.1016/S0140-6736(09)61460-4 PMC2810516

[8] Marmot M Allen J Boyce T , et al. Health equity in England: the Marmot Review 10 years on. London: Institute of Health Equity, 2020. 10.1136/bmj.m693 32094110

[9] Marmot Allen J Goldblatt P , et al. Build back fairer: the COVID-19 Marmot Review. The pandemic, socioeconomic and Health Inequalities in England. London: Institute of Health Equity, 2020.

[10] Lloyd-Williams M Shiels C Dowrick C , et al. Socio-economic deprivation and symptom burden in UK hospice patients with advanced cancer-findings from a longitudinal study. Cancers 2021; 13: 2537. 34064172 10.3390/cancers13112537 PMC8196745

[11] Malhotra C Krishnan A Yong JR , et al. Socio-economic inequalities in suffering at the end of life among advanced cancer patients: results from the APPROACH study in five Asian countries. Int J Equity Health 2020; 19: 158. 32912232 10.1186/s12939-020-01274-5 PMC7488341

[12] Delgado-Guay M Ferrer J Rieber AG , et al. Financial distress and its associations with physical and emotional symptoms and quality of life among Advanced Cancer Patients. Oncologist 2015; 20: 1092–1098. 26205738 10.1634/theoncologist.2015-0026 PMC4571810

[13] Popovic M Lao N Bedard G , et al. Quality of life in patients with advanced cancer using the functional assessment of cancer therapy-general assessment tool: a literature review. World J Oncol 2013; 4: 8–17. 29147325 10.4021/wjon594w PMC5649914

[14] Pritchard S Cuvelier G Harlos M , et al. Palliative care in adolescents and young adults with cancer. Cancer 2011; 117(10 Suppl): 2323–2328. DOI: 10.1002/cncr.26044. PMID: 21523753; PMCID: PMC5231403. 21523753 10.1002/cncr.26044 PMC5231403

[15] Cook KA Bergeron K . Palliative care for young adults with life-limiting conditions: public health recommendations. BMJ Support Palliat Care 2020; 2020: bmjscare–2019. 10.1136/bmjspcare-2019-002042 PMC9304099 32561547

[16] Cockle-Hearne J Reed E Todd J , et al. The dying parent and dependent children: a nationwide survey of hospice and community palliative care support services. BMJ Support Palliat Care 2020; 2020: bmjspcare-2019-001947. 10.1136/bmjspcare-2019-001947 PMC9606526 32152037

[17] Bajwah S Edmonds P Yorganci E , et al. The association between ethnicity, socioeconomic deprivation and receipt of hospital-based palliative care for people with Covid-19: A dual centre service evaluation. Palliat Med 2021; 35: 1514–1518. 34098811 10.1177/02692163211022959

[18] Murtagh FE Ramsenthaler C Firth A , et al. A brief, patient- and proxy-reported outcome measure in advanced illness: validity, reliability and responsiveness of the Integrated Palliative care Outcome Scale (IPOS). Palliat Med 2019; 33: 1045–1057. 31185804 10.1177/0269216319854264 PMC6691591

[19] McLennan D Noble S Noble M , et al. The English indices of deprivation 2019. Technical report. Ministry of Housing, Communities and Local Government; 2019.

[20] Fei F Siegert RJ Zhang X , et al. Symptom clusters, associated factors and health-related quality of life in patients with chronic obstructive pulmonary disease: a structural equation modelling analysis. J Clin Nurs. Epub ahead of print 30 January 2022. DOI: 10.1111/jocn.16234. PMC10078635 35098602

[21] Masso M Allingham SF Banfield M , et al. Palliative Care Phase: inter-rater reliability and acceptability in a national study. Palliat Med 2015; 29: 22–30. 25249239 10.1177/0269216314551814

[22] Abernethy AP Shelby-James T Fazekas BS , et al. The Australia-modified Karnofsky Performance Status (AKPS) scale: a revised scale for contemporary palliative care clinical practice [ISRCTN81117481]. BMC Palliat Care 2005; 4: 7–12. 16283937 10.1186/1472-684X-4-7 PMC1308820

[23] Hoffmann JP . Regression models for categorical, count, and related variables. Berkeley, CA: University of California Press, 2016.

[24] Cohen J . Statistical Power Analysis for the Behavioral Sciences. 2nd ed. New York: Routledge, 1988.

[25] Davies JM Maddocks M Chua K-C , et al. Socioeconomic position and use of hospital-based care towards the end of life: a mediation analysis using the English Longitudinal Study of Ageing. Lancet Public Health 2021; 6: e155–e163. 10.1016/S2468-2667(20)30292-9 PMC7910274 33571459

[26] Gott M Morgan T Williams L . Gender and palliative care: a call to arms. Palliat Care Soc Pract 2020; 14: 2632352420957997. 33134926 10.1177/2632352420957997 PMC7576896

[27] White IR Royston P Wood AM . Multiple imputation using chained equations: Issues and guidance for practice. Stat Med 2011; 30: 377–399. 21225900 10.1002/sim.4067

[28] Plumpton CO Morris T Hughes DA , et al. Multiple imputation of multiple multi-item scales when a full imputation model is infeasible. BMC Res Notes 2016; 9: 45–45. 26809812 10.1186/s13104-016-1853-5 PMC4727289

[29] Kleinke K . Multiple imputation under violated distributional assumptions: a systematic evaluation of the assumed robustness of predictive mean matching. J Educ Behav Stat 2017; 42: 371–404.

[30] Graham JW Olchowski AE Gilreath TD . How many imputations are really needed? Some practical clarifications of multiple imputation theory. Prev Sci 2007; 8: 206–213. 17549635 10.1007/s11121-007-0070-9

[31] van Ginkel JR Linting M Rippe RCA , et al. Rebutting existing misconceptions about multiple imputation as a method for handling missing data. J Pers Assess 2020; 102: 297–308. 30657714 10.1080/00223891.2018.1530680

[32] Linden A Mathur MB VanderWeele TJ . Conducting sensitivity analysis for unmeasured confounding in observational studies using E-values: the evalue package. Stata J 2020; 20: 162–175.

[33] Westreich D Greenland S . The Table 2 fallacy: Presenting and Interpreting Confounder and modifier coefficients. Am J Epidemiol 2013; 177: 292–298. 23371353 10.1093/aje/kws412 PMC3626058

[34] Finucane AM Swenson C MacArtney JI , et al. What makes palliative care needs “complex”? A multisite sequential explanatory mixed methods study of patients referred for specialist palliative care. BMC Palliat Care 2021; 20: 18. 33451311 10.1186/s12904-020-00700-3 PMC7809819

[35] The vicious cycle of fuel poverty and terminal illness. Marie Curie Cancer Care, https://www.mariecurie.org.uk/globalassets/media/documents/policy/policy-publications/2020/fuel-poverty-and-terminal-illness.pdf (2020, accessed 19 February 2022).

[36] Gardiner C Robinson J Connolly M , et al. Equity and the financial costs of informal caregiving in palliative care: a critical debate. BMC Palliat Care 2020; 19: 71. 32429888 10.1186/s12904-020-00577-2 PMC7236957

[37] Gott M Allen R Moeke-Maxwell T , et al. ‘No matter what the cost’: a qualitative study of the financial costs faced by family and whānau caregivers within a palliative care context. Palliat Med 2015; 29: 518–528. 25680378 10.1177/0269216315569337 PMC4441882

[38] Lewis JM DiGiacomo M Currow DC , et al. Dying in the margins: understanding palliative care and socioeconomic deprivation in the developed world. J Pain Symptom Manag 2011; 42: 105–118. 10.1016/j.jpainsymman.2010.10.265 21402460

[39] Curie M . Dying in Poverty: Exploring poverty at the end of life in the UK, https://www.mariecurie.org.uk/globalassets/media/documents/policy/dying-in-poverty/h420-dying-in-poverty-5th-pp.pdf (2022, accessed 27 May 2022).

[40] Hussain JA Koffman J Bajwah S . Invited editorials. Palliat Med 2021; 35: 810–813. 33957826 10.1177/02692163211012887

[41] Marmot M . The health gap: the challenge of an unequal world. Lancet 2015; 386: 2442–2444. 26364261 10.1016/S0140-6736(15)00150-6

[42] Yadav K Lewis RJ . Immortal time bias in observational studies. JAMA 2021; 325: 686–687. 33591334 10.1001/jama.2020.9151

[43] National End of Life Care Inelligence Network. Deprivation and death: variation in place and cause of death. London: National End of Life Care Inelligence Network, 2012.

[44] Chukwusa E Yu P Verne J , et al. Regional variations in geographic access to inpatient hospices and place of death: a population-based study in England, UK. PLoS One 2020; 15: e0231666–e0231666. 10.1371/journal.pone.0231666 PMC7164606 32302344

[45] Small N Gardiner C Barnes S , et al. “You get old, you get breathless, and you die”: chronic obstructive pulmonary disease in Barnsley, UK. Health Place 2012; 18: 1396–1403. 22889997 10.1016/j.healthplace.2012.07.004

[46] White C . Just 4% of UK doctors come from working class backgrounds. BMJ 2016; 355: i6330.

[47] Snee H Goswami H . Who cares? Social mobility and the ‘Class Ceiling’ in Nursing. Sociol Res Online 2021; 26: 562–580.

[48] Smith P Johnson CS Ariti C , et al. Focus on: Hospital admissions from care homes. The Health Foundation Quality Watch, https://wwwhealthorguk/sites/default/files/QualityWatch_FocusOnHospitalAdmissionsFromCareHomespdf (2015, accessed 19 May 2022).

[49] Kelfve S Wastesson J Fors S , et al. Is the level of education associated with transitions between care settings in older adults near the end of life? A nationwide, retrospective cohort study. Palliat Med 2018; 32: 366–375. 28952874 10.1177/0269216317726249

[50] Grundy E Holt G . The socioeconomic status of older adults: how should we measure it in studies of health inequalities? J Epidemiol Community Health 2001; 55: 895–904. 11707484 10.1136/jech.55.12.895 PMC1731799

[51] Mathur R Bhaskaran K Chaturvedi N , et al. Completeness and usability of ethnicity data in UK-based primary care and hospital databases. J Public Health 2014; 36: 684–692. 10.1093/pubmed/fdt116 PMC4245896 24323951

