# Supplementary material for: The association between socioeconomic position and the symptoms and concerns of hospital inpatients seen by specialist palliative care: Analysis of routinely collected patient data
Source: Palliat Med. 2022 Aug 10;37(4):575–85. doi: 10.1177/02692163221115331 (PMC10074756; doi:10.1177/02692163221115331)
Supplement: sj-pdf-1-pmj-10.1177_02692163221115331 – Supplemental material for The association between socioeconomic position and the symptoms and concerns of hospital inpatients seen by specialist palliative care: Analysis of routinely collected patient data [file sj-pdf-1-pmj-10.1177_02692163221115331.pdf]

Supplementary material

Table 1: National distribution of deaths by deprivation quintile for all deaths in people aged ≥60 years old, registered in England in 2019

IMD quintile	n	%
1	85331	19.3
2	87046	19.7
3	92109	20.8
4	92310	20.9
5	85992	19.4
Total	442788	100%

Data available here, accessed: 17.02.22:
https://www.ons.gov.uk/peoplepopulationandcommunity/birthsdeathsandmarriages/deaths/adhocs/12413deathregistrationsandpopulationsbyindexofmultipledeprivationimddecileenglandandwales2019

Table 2: National distribution of deaths by underlying cause of death for all deaths in people aged ≥60 years old, registered in England and Wales in 2019

Cause of death	N	%
cancer	132857	28.1
dementia	66332	14.0
cardiovascular	118787	25.1
respiratory	68821	14.5
other	86476	18.3
	473273	100%

Data available here, accessed: 17.02.22:
https://www.ons.gov.uk/peoplepopulationandcommunity/birthsdeathsandmarriages/deaths/datasets/the21stcenturymortalityfilesdeathsdataset

Table 3: distribution of age, IMD, diagnosis, and IPOS subscale scores by hospital site

	**hospital 1**	**hospital 2**
n	4392	3468
Age, median (IQR)	84 (77, 90)	79 (70, 86)
IMD q1 (most deprived)	377 (8.6%)	768 (22.1%)
q2	515 (11.7%)	1260 (36.3%)
q3	537 (12.2%)	781 (22.5%)
q4	1413 (32.2%)	440 (12.7%)
q5	1550 (35.3%)	219 (6.3%)
cancer	1660 (38.7%)	1353 (39.9%)
dementia	392 (9.1%)	250 (7.4%)
cardiovascular	697 (16.2%)	654 (19.3%)
respiratory	247 (5.8%)	213 (6.3%)
other	1297 (30.2%)	925 (27.2%)
physical subscale mean (SD) (n non missing)	8.6 (6.6) (n=3276)	10.3 (6.0) (n=1607)
emotional subscale mean (SD) (n non missing)	4.5 (3.5) (n=2807)	5.7 (3.9) (n=1883)
practical subscale mean (SD) (n non missing)	5.7 (3.5) (n=3061)	4.1 (3.6) (n=1900)

Table 4: Reason for referral to specialist palliative care

**Reason**	**N**	**%**
Pain or other physical symptoms	2679	34.08
Terminal Care	2480	31.55
Palliative care opinion	1338	17.02
Other	487	6.20
Discharge Advice/Planning	344	4.38
Family carers support	314	3.99
Psychological support	186	2.37
Missing	23	0.29
Palliative rehabilitation	9	0.11
Total	7860	100

Figure 1: Proportion of patients with moderate, severe, or overwhelming* on each of the IPOS items (using complete case data)

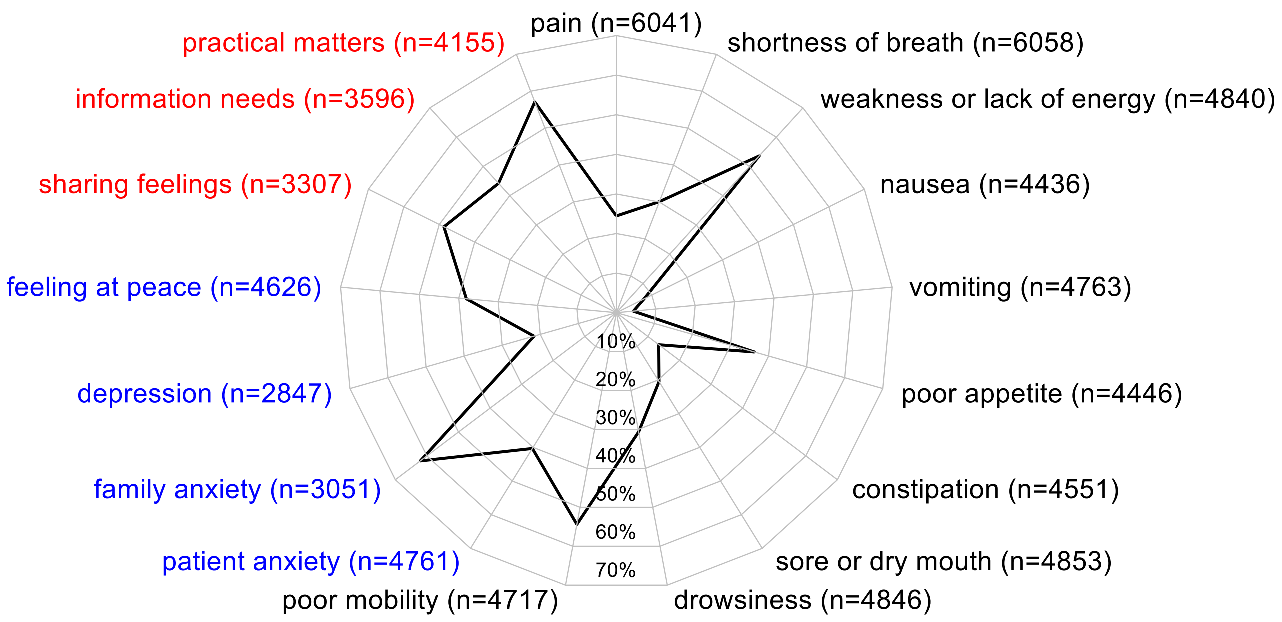

*'Moderate, severe, or overwhelming' represent high scores (2-4, on the 0-4 IPOS scale), with higher scores representing worse symptoms and problems. Items in the emotional and practical subscales have different text associated with the scale, for full details see the IPOS measure: https://pos-pal.org/

Text colour indicates grouping of items in the three subscales: black (physical) blue (emotional) red (communication/practical)

Table 5: distribution of IPOS scores

	% 2-4	% 0	% 1	% 2	% 3	% 4
pain	24.4	50.8	24.8	18.7	4.9	0.8
shortness of breath	30.1	44.6	25.3	20.9	7.7	1.4
weakness or lack of energy	53.6	30.2	16.2	23.1	18.7	11.9
nausea	7.8	84.5	7.7	5.6	1.9	0.3
vomiting	4.3	91.6	4.1	2.8	1.2	0.3
poor appetite	36.2	46.2	17.5	21.3	11.1	3.9
constipation	13.4	75.2	11.4	9.7	3.3	0.5
sore or dry mouth	20.5	46.2	33.3	15.9	3.7	0.8
drowsiness	30.6	51.6	17.7	13.0	8.0	9.6
poor mobility	54.4	31.9	13.8	17.7	17.9	18.8
patient anxiety	40.3	34.3	25.4	28.6	10.0	1.8
family anxiety	62.1	14.7	23.2	36.5	20.4	5.2
depression	21.6	61.1	17.2	16.2	4.6	0.9
feeling at peace	38.1	23.5	38.4	24.9	7.8	5.4
sharing feelings	48.7	26.0	25.3	18.0	9.5	21.3
information needs	44.2	29.4	26.4	18.5	7.3	18.4
practical matters	57.1	22.9	20.0	29.2	12.7	15.2

Table 6: Odds of having missing data (after median imputation) for the three IPOS subscales (n=6266)

	physical		emotional		Communication/practical	
	OR	CI	OR	CI	OR	CI
**Age**	0.99	(0.99 - 1.00)	1.00	(0.99 - 1.01)	1.01	(1.00 - 1.02)
**Gender**						
men	ref	ref	ref	ref	ref	ref
women	1.02	(0.89 - 1.17)	1.10	(0.97 - 1.24)	1.09	(0.95 - 1.24)
**Total clinical time with patient during episode***						
0-20 minutes	1.46	(1.19 - 1.79)	1.65	(1.36 - 1.99)	1.34	(1.09 - 1.64)
25-45 minutes	1.37	(1.14 - 1.65)	1.32	(1.10 - 1.59)	1.30	(1.07 - 1.57)
50-85 minutes	1.24	(1.02 - 1.49)	1.12	(0.93 - 1.35)	1.14	(0.94 - 1.39)
90-500 minutes	ref	ref	ref	ref	ref	ref
**IMD**						
q1 (most deprived)	1.37	(1.06 - 1.78)	0.98	(0.79 - 1.23)	1.09	(0.85 - 1.38)
q2	1.18	(0.92 - 1.51)	0.97	(0.79 - 1.19)	1.01	(0.81 - 1.27)
q3	1.40	(1.08 - 1.80)	0.99	(0.80 - 1.22)	1.20	(0.95 - 1.51)
q4	1.41	(1.11 - 1.80)	0.92	(0.76 - 1.11)	1.10	(0.89 - 1.35)
q5	ref	ref	ref	ref	ref	ref
**Ethnicity**						
white british	ref	ref	ref	ref	ref	ref
white other	1.29	(0.97 - 1.71)	1.12	(0.84 - 1.50)	1.17	(0.86 - 1.58)
black	1.21	(0.98 - 1.51)	1.50	(1.19 - 1.89)	1.24	(0.98 - 1.57)
asian	0.99	(0.66 - 1.48)	1.10	(0.74 - 1.64)	1.07	(0.71 - 1.62)
other	1.11	(0.78 - 1.58)	1.42	(0.99 - 2.04)	1.28	(0.88 - 1.85)
missing	1.20	(0.97 - 1.49)	1.22	(0.98 - 1.51)	1.46	(1.17 - 1.82)
**Living alone**						
not living alone	ref	ref	ref	ref	ref	ref
lives alone	0.89	(0.75 - 1.04)	0.97	(0.83 - 1.12)	0.93	(0.79 - 1.09)
missing	1.03	(0.85 - 1.25)	1.41	(1.14 - 1.73)	1.47	(1.19 - 1.81)
**Diagnosis**						
cancer	ref	ref	ref	ref	ref	ref
dementia	1.51	(1.15 - 1.99)	2.12	(1.67 - 2.68)	1.49	(1.15 - 1.92)
cardiovascular	1.34	(1.08 - 1.66)	1.76	(1.45 - 2.14)	1.30	(1.05 - 1.60)
respiratory	1.11	(0.83 - 1.49)	0.73	(0.53 - 1.00)	0.76	(0.55 - 1.06)
other	0.96	(0.80 - 1.17)	1.19	(0.99 - 1.42)	1.05	(0.87 - 1.27)
**Phase of illness**						
unstable	ref	ref	ref	ref	ref	ref
stable	1.00	(0.71 - 1.41)	0.84	(0.56 - 1.26)	0.73	(0.47 - 1.15)
deteriorating	0.80	(0.66 - 0.98)	1.05	(0.88 - 1.26)	1.07	(0.88 - 1.30)
dying	1.16	(0.95 - 1.41)	1.06	(0.88 - 1.27)	1.35	(1.11 - 1.63)
**AKPS**	0.98	(0.97 - 0.98)	0.95	(0.95 - 0.96)	0.95	(0.95 - 0.96)
**Hospital site**						
hospital 1	ref	ref	ref	ref	ref	ref
hospital 2	9.69	(8.10 - 11.61)	2.18	(1.86 - 2.55)	3.90	(3.30 - 4.62)

*includes all time a clinician spends with the patient, does not include clinician time spent with family, other healthcare professionals, or admin time

Table 7: Missing values on covariates, for cases with non-missing subscale data (after median imputation)

	Complete subscale data		
	physical	emotional	practical
n (%)	4883	4690	4961
diagnosis missing	50 (1.0%)	46 (1.0%)	49 (1.0%)
phase missing	7 (0.1%)	9 (0.2%)	10 (0.2%)
akps missing	23 (0.5%)	13 (0.3%)	22 (0.4%)
ethnicity	589 (12.1%)	557 (11.9%)	559 (11.3%)
living alone	705 (14.4%)	672 (14.3%)	684 (13.8%)

Table 8: Main models, full results for all covariates (corresponds to figure 1 and table 4 in the manuscript)

	Physical n=4803		Emotional n=4622		Practical n=4880	
age	-0.03	[-0.06,-0.01]	-0.07	[-0.08,-0.05]	0.00	[-0.01,0.02]
women (ref)	-	-	-	-	-	-
men	0.10	[-0.26,0.46]	-0.03	[-0.23,0.18]	0.01	[-0.19,0.21]
imd q1 (most deprived)	-0.51	[-1.15,0.12]	-0.14	[-0.50,0.23]	0.56	[0.21,0.91]
imd q2	-0.33	[-0.91,0.26]	-0.16	[-0.50,0.17]	0.33	[0.01,0.64]
imd q3	-0.05	[-0.65,0.56]	0.17	[-0.17,0.52]	0.44	[0.12,0.76]
imd q4	-0.29	[-0.79,0.22]	0.11	[-0.19,0.40]	0.34	[0.07,0.61]
imd q5 (ref)	-	-	-	-	-	-
white British (ref)	-	-	-	-	-	-
white other	0.22	[-0.66,1.11]	0.42	[-0.06,0.90]	-0.14	[-0.58,0.29]
black	-0.23	[-0.92,0.47]	-0.47	[-0.90,-0.05]	0.15	[-0.27,0.56]
asian	0.23	[-0.96,1.43]	-0.19	[-0.79,0.41]	-0.09	[-0.76,0.59]
other	-0.82	[-1.94,0.29]	0.03	[-0.76,0.81]	0.17	[-0.55,0.88]
missing	1.01	[0.31,1.70]	0.57	[0.14,1.01]	-0.24	[-0.67,0.18]
not living alone (ref)	-	-	-	-	-	-
lives alone	0.42	[0.01,0.83]	0.20	[-0.03,0.44]	0.11	[-0.11,0.33]
missing	0.08	[-0.57,0.72]	-0.09	[-0.49,0.32]	0.19	[-0.21,0.58]
cancer (ref)	-	-	-	-	-	-
dementia	-0.95	[-1.76,-0.14]	-0.63	[-1.10,-0.17]	0.75	[0.29,1.20]
cardiovascular	-1.49	[-2.09,-0.90]	-0.53	[-0.88,-0.19]	0.40	[0.07,0.74]
respiratory	-0.89	[-1.63,-0.15]	0.33	[-0.12,0.78]	-0.25	[-0.63,0.13]
other	-1.98	[-2.48,-1.48]	-0.31	[-0.60,-0.01]	0.32	[0.04,0.60]
stable	-2.51	[-3.19,-1.83]	-1.49	[-1.99,-0.99]	0.11	[-0.32,0.54]
unstable (ref)	-	-	-	-	-	-
deteriorating	-0.89	[-1.31,-0.47]	-0.85	[-1.12,-0.58]	-0.28	[-0.52,-0.03]
dying	-0.53	[-1.08,0.01]	-1.17	[-1.49,-0.86]	-0.64	[-0.95,-0.34]
AKPS	-0.08	[-0.10,-0.07]	-0.02	[-0.02,-0.01]	-0.04	[-0.05,-0.04]
Hospital 1 (ref)	-	-	-	-	-	-
Hospital 2	1.86	[1.39,2.32]	0.76	[0.50,1.03]	-1.54	[-1.79,-1.29]

Table 9: Model fit statistics and moderation effects

Model 1: Minimally adjusted model, adjusted for **age, sex, hospital site, IMD**
Model 2: Fully adjusted model, model 1 + **ethnicity, lives alone, diagnosis, Phase, AKPS**
Model 3: Moderation by age, model 2 + **age##IMD**
Model 4: Moderation by sex, model 2 + **sex##IMD**

	**Model 1**<br><br>**minimally adjusted model**	**Model 2**<br><br>**fully adjusted model**	**Model 3**<br><br>**moderation by age**	**Model 4**<br><br>**moderation by sex**
**Physical (n=4803)**				
**IMD q1 (most deprived)**	-0.59 [-1.23,0.05]	-0.51 [-1.15,0.12]	-0.01 [-0.07,0.05]	-0.63 [-1.82,0.55]
**q2**	-0.30 [-0.89,0.29]	-0.33 [-0.91,0.26]	-0.02 [-0.08,0.03]	0.05 [-0.99,1.10]
**q3**	-0.02 [-0.63,0.60]	-0.05 [-0.65,0.56]	0.00 [-0.06,0.06]	0.65 [-0.50,1.80]
**q4**	-0.29 [-0.80,0.22]	-0.29 [-0.79,0.22]	-0.07 [-0.13,-0.02]	0.79 [-0.22,1.80]
**q5**	ref	ref	ref	ref
R^2	0.0178	0.0634	0.0651	0.0647
ΔR^2	0.0178	0.0020	0.0018	0.0013
F	13.40	2.36	2.23	1.73
p	<0.0001	0.0380	0.0630	0.1404
**Emotional (n=4622)**				
**IMD q1 (most deprived)**	-0.21 [-0.57,0.16]	-0.14 [-0.50,0.23]	-0.01 [-0.04,0.03]	-0.44 [-1.12,0.24]
**q2**	-0.24 [-0.57,0.09]	-0.16 [-0.50,0.17]	0.00 [-0.04,0.03]	-0.47 [-1.08,0.13]
**q3**	0.17 [-0.18,0.52]	0.17 [-0.17,0.52]	-0.01 [-0.04,0.03]	-0.54 [-1.20,0.13]
**q4**	0.10 [-0.19,0.40]	0.11 [-0.19,0.40]	-0.03 [-0.06,0.00]	-0.10 [-0.68,0.48]
**q5**	ref	ref	ref	ref
R^2	0.0689	0.0970	0.0977	0.0979
ΔR^2	0.0689	0.0036	0.0007	0.0009
F	48.32	3.39	0.84	1.13
p	<0.0001	0.0047	0.4990	0.3419
**Practical (n=4880)**				
**IMD q1 (most deprived)**	0.67 [0.32,1.02]	0.56 [0.21,0.91]	-0.04 [-0.07,-0.01]	-0.21 [-0.87,0.45]
**q2**	0.44 [0.12,0.76]	0.33 [0.01,0.64]	-0.04 [-0.07,-0.01]	0.28 [-0.30,0.86]
**q3**	0.45 [0.12,0.77]	0.44 [0.12,0.76]	-0.03 [-0.06,0.00]	-0.09 [-0.71,0.52]
**q4**	0.41 [0.13,0.68]	0.34 [0.07,0.61]	0.00 [-0.03,0.03]	0.15 [-0.39,0.69]
**q5**	ref	ref	ref	ref
R^2	0.0545	0.0918	0.0941	0.0923
ΔR^2	0.0545	0.0006	0.0023	0.0005
F	39.90	0.56	3.09	0.65
p	<0.0001	0.7337	0.0150	0.6264

Interpretation:
For model 1, model fit comparison is against the null model, for model 3 and 4 comparison is against model 2
For model 3 and 4, IMD coefs are for the interaction effects e.g. age##imd (interpreted as the difference in age slopes for IMD q1/4 compared to q5) and sex##imd (interpreted as the effect of being male versus female in IMD q1/4 versus q5)
R^2 is the proportion of the variance in the dependent variable predicted from the independent variables
ΔR^2 is the increase in R^2 between models
F is based on the Wald statistic and sums the predictive power of the block of independent variables (blocks of variables indicated in bold at the top of the table) and p tests the overall significance of the predictive power of the block.

Table 10: Sensitivity analysis; coefficients for IMD in the final model and in the complete case analysis, models adjusted for age, sex, ethnicity, living alone, diagnosis, AKPS, Phase, hospital site

	final model	complete case	MI
**Physical n**	4803	3805	7310*
**IMD q1 (most deprived)**	-0.51 [-1.15,0.12]	-0.20 [-0.80,0.41]	-0.44 [-1.02,0.15]
**q2**	-0.33 [-0.91,0.26]	-0.39 [-0.94,0.15]	-0.31 [-0.83,0.21]
**q3**	-0.05 [-0.65,0.56]	0.23 [-0.34,0.80]	0.10 [-0.42,0.63]
**q4**	-0.29 [-0.79,0.22]	-0.16 [-0.63,0.31]	-0.30 [-0.75,0.15]
**q5**	ref	ref	ref
**Emotional n**	4622	1767	
**IMD q1 (most deprived)**	-0.14 [-0.50,0.23]	-0.33 [-0.86,0.20]	-0.21 [-0.54,0.12]
**q2**	-0.16 [-0.50,0.17]	-0.02 [-0.53,0.49]	-0.08 [-0.36,0.21]
**q3**	0.17 [-0.17,0.52]	0.04 [-0.47,0.55]	0.15 [-0.16,0.46]
**q4**	0.11 [-0.19,0.40]	0.16 [-0.27,0.59]	0.12 [-0.13,0.37]
**q5**	ref	ref	ref
**Practical n**	4880	2600	
**IMD q1 (most deprived)**	0.56 [0.21,0.91]	0.63 [0.21,1.05]	0.63 [0.29,0.97]
**q2**	0.33 [0.01,0.64]	0.42 [0.05,0.78]	0.35 [0.01,0.69]
**q3**	0.44 [0.12,0.76]	0.26 [-0.11,0.63]	0.35 [0.04,0.65]
**q4**	0.34 [0.07,0.61]	0.39 [0.09,0.69]	0.35 [0.09,0.61]
**q5**	ref	ref	ref

*missing data on the categorical variables gender, diagnosis and Phase were not imputed to ensure the MI model would converge, therefore the n is less than the total sample N of 7860. Missing data for age and AKPS were imputed as part of the MI model.
